# Case report: Primary sarcoma of the mandible with a novel *SLMAP-BRAF* fusion

**DOI:** 10.3389/fonc.2024.1369046

**Published:** 2024-03-28

**Authors:** Peng Zhou, Wei Liu, Jiaoyun Zheng, Haixia Zhang, Jiadi Luo

**Affiliations:** ^1^ Department of Pathology, The Second Xiangya Hospital, Central South University, Changsha, Hunan, China; ^2^ Hunan Clinical Medical Research Center for Cancer Pathogenic Genes Testing and Diagnosis, Changsha, Hunan, China; ^3^ Beijing Novogene Bioinformatics Technology Co., Ltd., Beijing, China; ^4^ Department of Oncology, The Second Xiangya Hospital, Central South University, Changsha, Hunan, China

**Keywords:** sarcoma, BRAF rearrangement, SLMAP, jaw bone tumor, NGS

## Abstract

Primary sarcomas of the jaw are very rare tumor with unclear mechanism of tumorigenesis. Identification of genetic alterations contributes to better understanding of tumorigenesis and extension of tumor spectrum, as well as potential therapeutic targets application. Herein, we firstly report a case of primary sarcoma in the mandible with novel *SLMAP-BRAF* fusion. Morphologically, the tumor was composed of histiocyte-like cells, larger epithelioid cells, spindle cells and osteoclast-like giant cells with moderate atypia. Focally, it mimicked tenosynovial giant cell tumor or biphasic synovial sarcoma, and even giant cell tumor of bone. SATB2 was diffusely expressed, while p63 and p16 were locally positive with loss expression of p16 in histiocyte-like and larger epithelioid cells. *SLMAP-BRAF* (S11:B10) fusion was detected by both DNA and RNA NGS, and further verified by sanger sequencing, DNA electrophoresis and FISH. Then a descriptive diagnosis of *BRAF* rearrangement sarcoma with moderate-grade malignancy (non-specific type) was given according to the biological behavior, morphological features and gene alteration. The patient finished six cycles of chemotherapy after hemimaxillectomy. Within 7 months of follow-up, no tumor recurrence or metastasis was observed. Our case has enriched the spectrum of jaw bone tumor and *BRAF* rearrangement tumor.

## Introduction

Primary sarcomas of the jaw bone, mainly including osteosarcomas, are a series of very rare lesions. Clinically, maxillofacial swelling and pain are often the first symptoms. From morphological and immunological observation to genetic change information, the spectrum of jaw tumors has been further expanded. For example, in the fifth edition of WHO head and neck tumors, rhabdomyosarcoma with TFCP2 gene rearrangement was added to the malignant jaw tumor section (1).

Gene rearrangement plays a decisive role in the occurrence of various tumors, and nomenclature of sarcomas by gene rearrangement applies in pathological practice. The most famous one is NTRK rearrangement spindle cell tumor, mainly because of its effective therapeutic target (2). As it is well known, *BRAF* is a proto-oncogene, which is located on human chromosome 7 and encodes the RAF family serine/threonine protein kinase. This protein is involved in regulating the MAPK/ERK signaling pathway, affecting cell division, differentiation and secretion (3). There have been many reports on the correlation between *BRAF* mutations and multiple groups of malignant tumors (3). Recently, a few studies have found that *BRAF* gene fusion appears in different tumors. *BRAF* gene fusions occur in pilocytic astrocytoma (PA), which can be applied for the differential diagnosis of gliomas (4). In BRAF/RAS/NF1 triple wild-type melanomas, gene rearrangements are relatively enriched, including *BRAF* fusion (5). Victor L Quan team stated that *BRAF*-related fusions were responsible for 5% of Spitz neoplasms (6). *BRAF* fusions have been found in a series of myxoinflammatory fibroblastic sarcoma (MIFS) (7) and infantile fibrosarcoma (IFS) (8, 9). Besides, *BRAF* gene rearrangements have occasionally been reported in congenital mesoblastic nephroma (CMN) (10), prostate cancer (11), pheochromocytoma (12), and so on. Although gene fusion presents the generation of a “new” gene, functions of the protein encoded by the fusion gene are ultimately determined by fusion site and the master gene. So far, the common features of tumors harboring *BRAF* fusion gene haven’t been reported.

Sarcolemma associated protein gene (*SLMAP*), located in human chromosome 3, encodes a component of a conserved striatin interacting phosphatase and kinase complex, participating in a variety of cellular processes including cell cycle control, cell migration, golgi assembly, and apoptosis, and mutations in this gene are always associated with cardiac channelopathy (13). Literatures about *SLMAP* fusion are fairly rare, and usually with the label of “novel fusion”. For instance, novel *SLMAP-ALK* fusion was discovered in a patient with lung adenocarcinoma (14), novel *ERG-SLMAP* fusion in advanced prostate cancer (15), novel *SLMAP-NTRK2* in gangliogliomas (16). Two cases involving in *SLMAP-RAF1* fusion have been detected in spindle cell mesenchymal tumor with S100 and CD34 co-reactivity (17, 18).

In this article, we firstly present a case of primary sarcoma in the mandible with novel *SLMAP-BRAF* gene fusion, which broaden the spectrum of jaw tumor and *BRAF* rearrangement tumor.

## Case presentation

**Figure 1:**
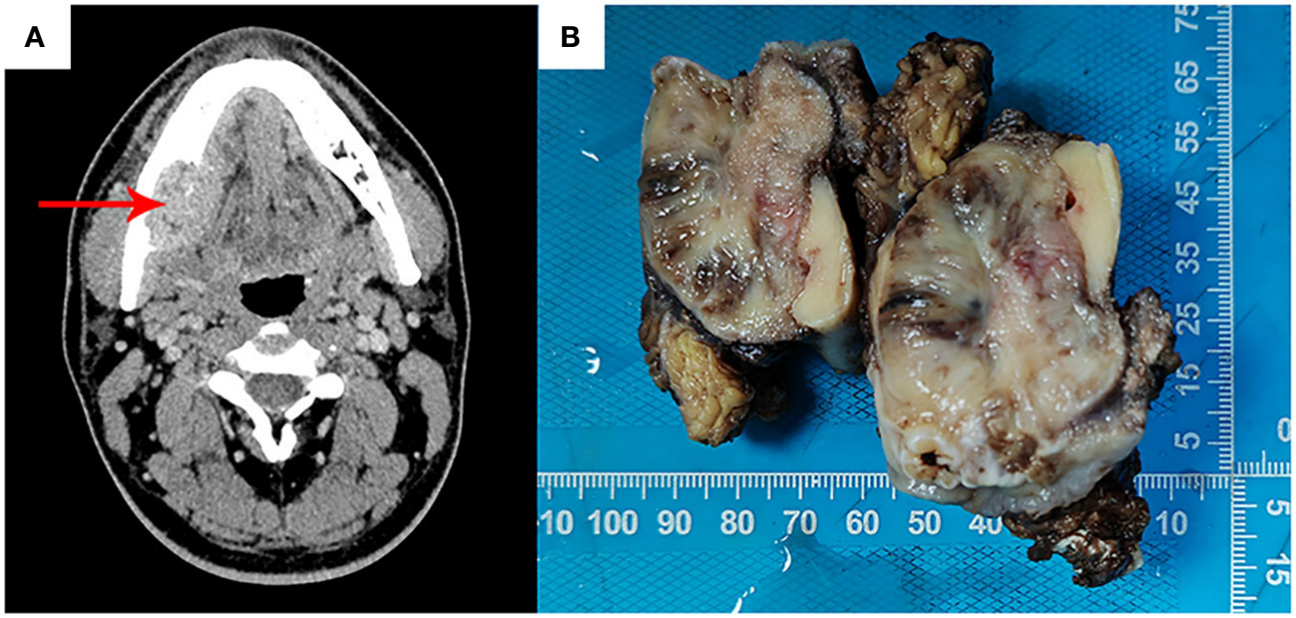
Axial CT image showed a mandible mass (red arrow), destroying bone cortex and extending into surrounding soft tissue, without periosteal reaction **(A)**. Gross specimen revealed a 5×4×3cm mass with a pale cut surface and focal hemorrhage **(B)**.

**Figure 2:**
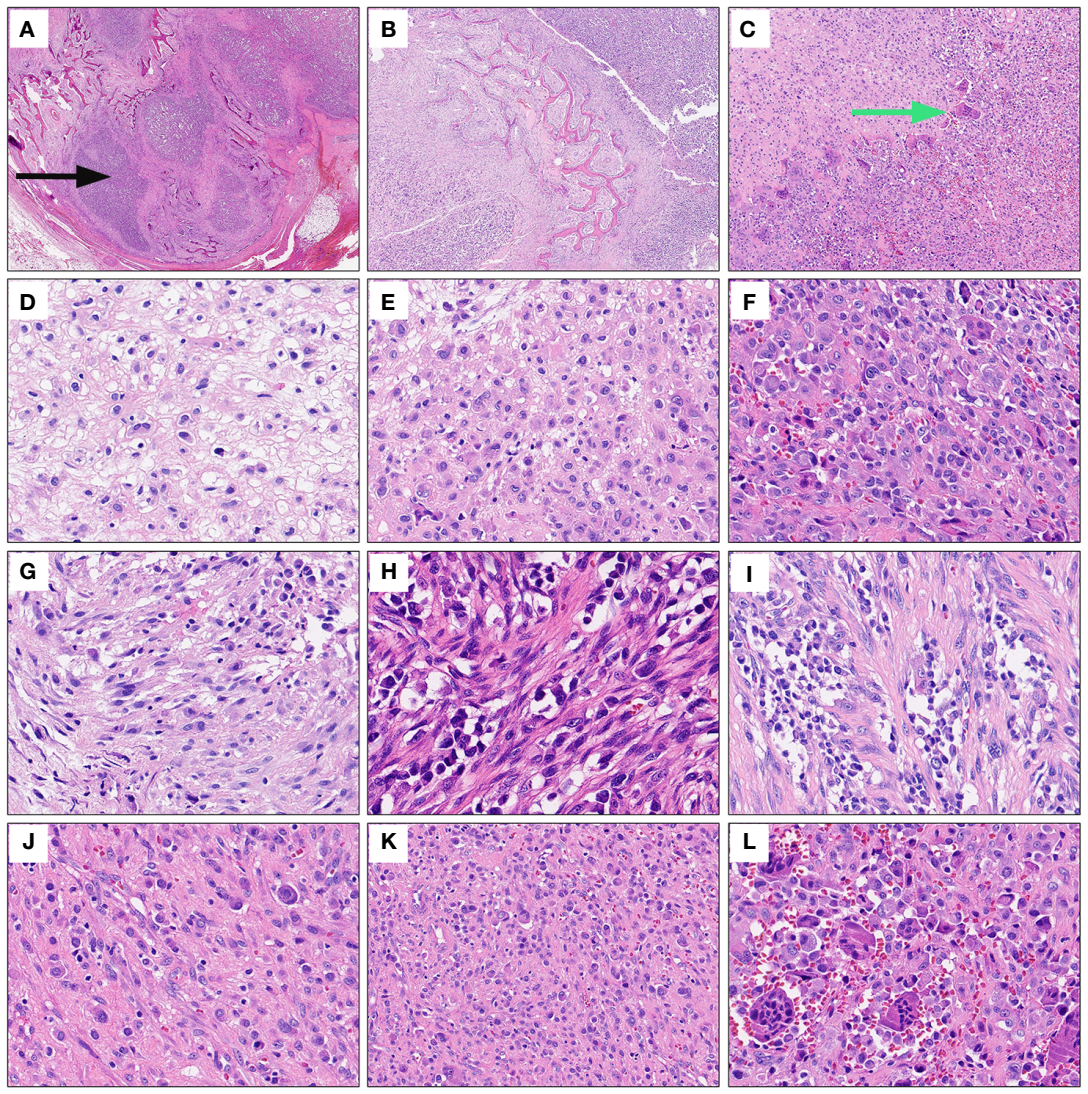
Representative morphological characteristics of the mandibular tumor. Under extra-low power field, H&E slides displayed a lobulated mandible mass destroying bone cortex (black arrows) **(A)** and intramedullary invasion **(B)**. Heterogeneous tumor cells accompanied with a few multinucleated giant cells under low power view (green arrows) **(C)**. Mononuclear tumor cells exhibited epithelioid feature with varying cell density **(D-F)**. Focally, epithelioid cells and spindle cells were present in varying proportions, forming biphasic structure **(G-I)**. Prominent pseudoglandular structure embedded in the spindle tumor cells **(H, I)**. The tumor was mainly composed of a mixture of histiocyte-like cells, larger epithelioid cells and osteoclast-like giant cells **(J–L)**. High power field exhibited two principal cell types: small histiocyte-like cells with pale cytoplasm and round or reniform nuclei, and larger epithelioid cells with amphophilic cytoplasm **(J, K)**. Scattered lymphocytes infiltration **(K)**. Numerous osteoclast-like multinucleated giant cells infiltrated focally **(L)**.

**Figure 3:**
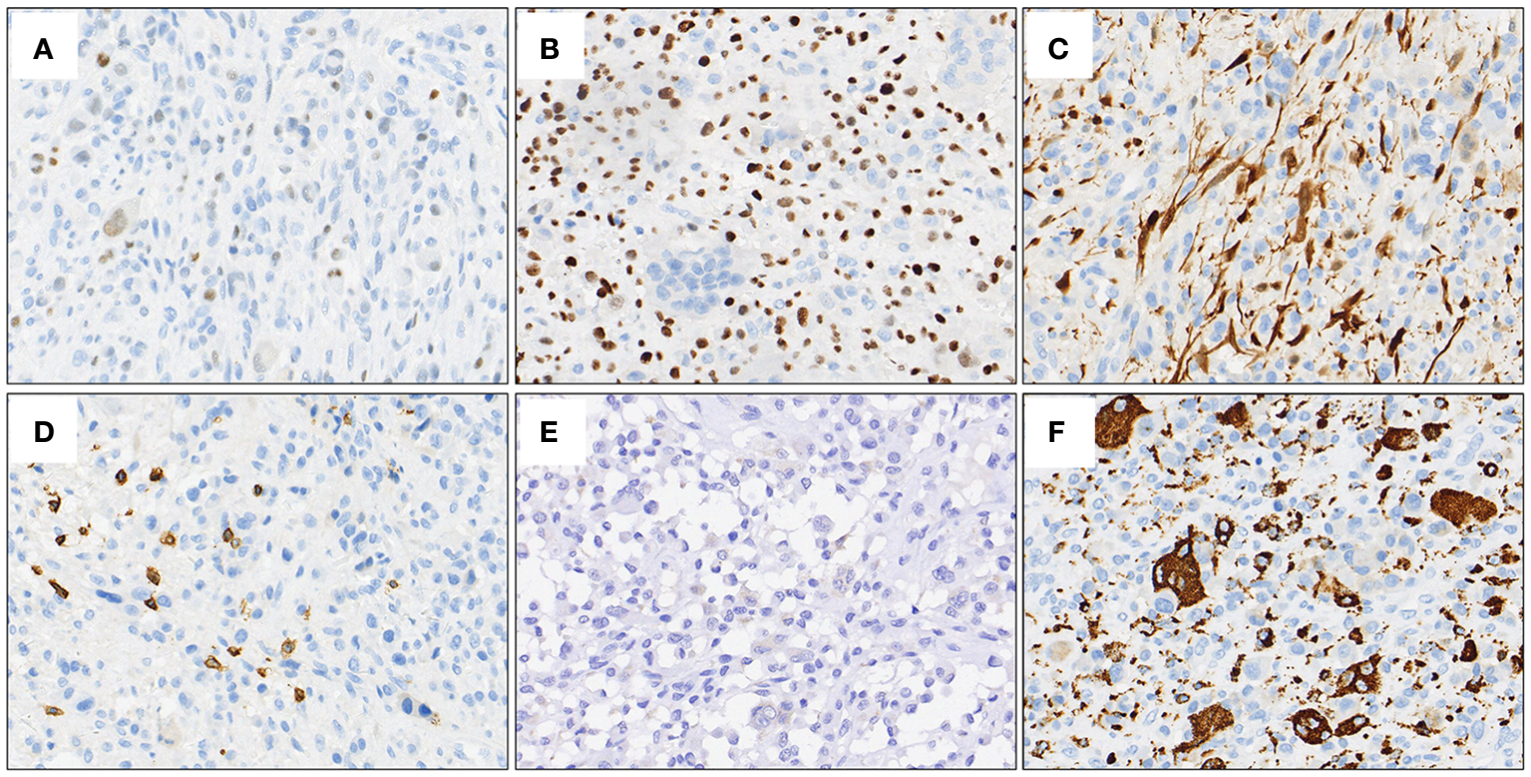
Representative IHC stains. Mononuclear tumor cells were positive for p63 **(A)** and SATB2 **(B)**, but dimly positive for BRAF **(E)**. P16 protein was strongly expressed in the spindle cells, while negative in mononuclear tumor cells **(C)**. Positive expression of CD3 demonstrated the infiltration of scattered T lymphocytes **(D)**. CD68 stain outlined scattered multinucleated giant cells **(F)**.

A 27-year-old male presented to the department of stomatology with mandible swelling. Axial CT image demonstrated a 5×3 cm mass in right mandible, extending into surrounding soft tissue (**Figure 1A**). After partial mandibulectomy, gross specimen revealed a 5×4×3cm mass with a pale cut surface and focal hemorrhage (**Figure 1B**). Morphologically, H&E slides showed a lobulated tumor destroying bone cortex (**Figure 2A**) and intramedullary invasion (**Figure 2B**). Heterogeneous tumor cells accompanied with a few multinucleated giant cells (**Figure 2C**). In some sections, mononuclear tumor cells exhibited epithelioid feature with varying cell density (**Figures 2D–F**). Focally, the tumor was consisted of biphasic structure, with varying proportions of epithelioid and spindle cells (**Figures 2G–I**). Prominent pseudoglandular structure embedded in the spindle tumor cells, which was similar to biphasic synovial sarcoma to some extent (**Figures 2H, I**). Generally, the tumor was composed of a mixture of histiocyte-like cells, larger epithelioid cells, and osteoclast-like giant cell, with moderate atypia (**Figures 2J–L**). Two principal cell types: small histiocyte-like cells with pale cytoplasm and round/reniform nuclei, and larger epithelioid cells with amphophilic cytoplasm, shared morphological overlap with tenosynovial giant cell tumor (**Figures 2J, K**). Scattered lymphocytes infiltration was captured (**Figure 2K**). Focally, numerous osteoclast-like multinucleated giant cells closely resembled the giant cell tumor of bone (**Figure 2L**). Moderate mitotic figures were seen (8/10HPF), and no necrosis was present. Immunohistochemical stains showed that mononuclear tumor cells were positive for p63 (**Figure 3A**) and SATB2 (**Figure 3B**), but dimly positive for BRAF (**Figure 3E**). P16 protein was strongly expressed in the spindle cells, while negative in mononuclear tumor cells (**Figure 3C**). CD3 stain revealed infiltration of scattered T lymphocytes (**Figure 3D**). Scattered multinucleated giant cells were positive for CD68 (**Figure 3F**). The Ki-67 proliferative index was up to 20%. The expressions of INI1 and H3K27me3 were retained, accompanied with normal P53 expression. Immunohistochemical stains performed negative for Desmin, S100, SOX-10, Clusterin, CD31, CD34, ERG, EMA, CK, Myogenin, H3G34W, H3K36M, HMB45 and Melan-A (not shown). According to the invasive biological behavior and histological features, low or moderate grade malignant mesenchymal tumor should be under consideration, although the histogenesis was unknown.

**Figure 4:**
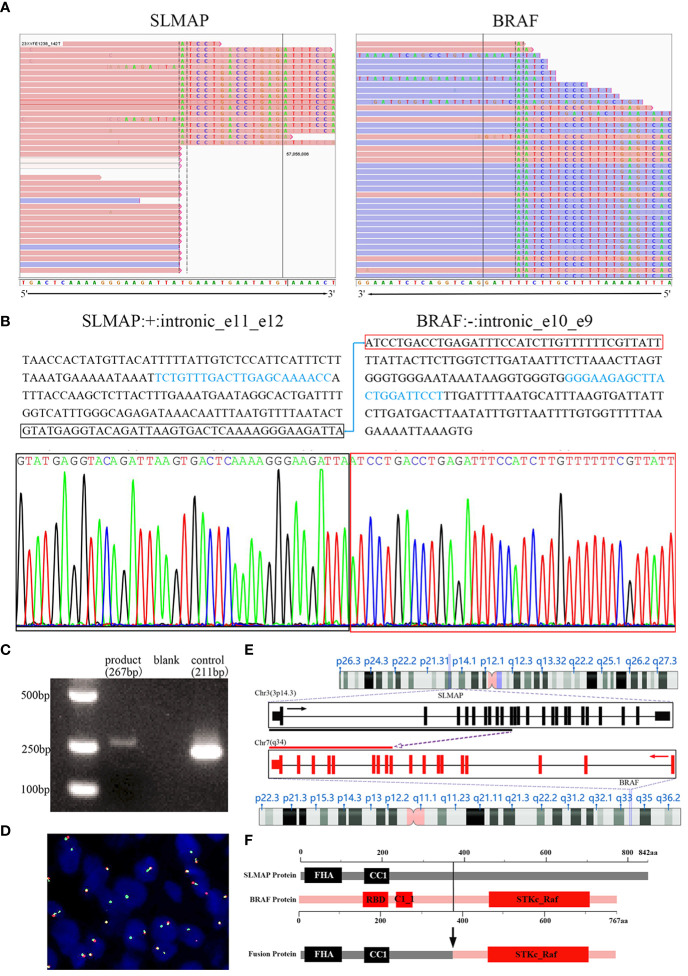
*SLMAP-BRAF* fusion was verified in the tumor. The IGV visualized map described the breakpoint site in the *SLMAP-BRAF* (S11:B10) fusion gene via NGS for the formalin-fixed paraffin-embedded tissue sample **(A)**. Sequences in intron 11 of *SLMAP* and intron 9 of *BRAF* were displayed. Proper PCR primers were then designed to amplify the fusion gene by PCR (sequences marked with light blue were the DNA fragments chosen for primer blast). The PCR product was then used for sanger sequencing and DNA gel electrophoresis. Sanger sequencing result confirmed the fusion site between *SLMAP* and *BRAF* **(B)**. DNA electrophoresis displayed a band slightly bigger than 250bp, which was consistent to the expected PCR product, a fragment of 267bp **(C)**. Break-apart FISH study with green-labeled probe located 3′ to *BRAF* and red-labeled probe located 5′ to *BRAF* showed an aberrant pattern of an isolated 3′ green signal accompanying with a normal juxtaposed red/green signal in 20% of interphase nuclei, implying an unbalanced rearrangement of *BRAF* gene **(D)**. Schematic of *SLMAP-BRAF* fusion **(E, F)**. In fusion gene, the exon 1-11 located 5′ of *SLMAP* and the exon 10-19 located 3′ of *BRAF* were retained **(E)**. The chimeric SLMAP-BRAF protein was composed of 767 amino acid residues, preserved the kinase domain and ATP binding pocket of BRAF, while the auto-inhibitory function domains of BRAF were lost, including the N-terminal RBD (Raf-like RAS-binding domain) and C1_1 (Phorbol esters/diacylglycerol binding domain) **(F)**.

Subsequently, molecular analyses were employed using DNA- and RNA-based NGS methods. *SLMAP-BRAF* fusion was detected by both DNA and RNA sequencing techniques. The IGV visualized map described the breakpoint site in the *SLMAP-BRAF* (S11:B10) fusion gene (**Figure 4A**). Further molecular validations were successfully achieved (**Figures 4B–D**). DNA sequences of the intronic breakpoint site between exon 11 and exon 12 of *SLMAP* (NM_001304420.3: +: intronic_e11_e12), as well as intronic sequences between exon 10 and exon 9 of *BRAF* (NM_004333.4: -: intronic_e10_e9) were extracted separately, proper primers were then designed to amplify the fusion gene by PCR (*SLMAP-BRAF* Forward primer: TCTGTTTGACTTGAGCAAAACC; *SLMAP-BRAF* Reverse primer: AGGAATCCAGTAAGCTCTTCCC). The PCR product was subsequently used for sanger sequencing and DNA gel electrophoresis. Sanger sequencing result verified *SLMAP-BRAF* fusion (**Figure 4B**). Meanwhile, DNA electrophoresis displayed a band slightly bigger than 250bp, which was consistent to the expected PCR product, a fragment of 267bp (**Figure 4C**). FISH visually exhibited an unbalanced rearrangement of *BRAF* (**Figure 4D**). In this fusion related chimeric gene (**Figure 4E**), the kinase domain and ATP binding pocket of *BRAF* were preserved, while the auto-inhibitory function domains of *BRAF* were lost (**Figure 4F**).

According to diagnostic criteria of French Fédération Nationale des Centres de Lutte Contre le Cancer (FNCLCC), the tumor should be classified as grade 2: no exactly histological type, mitotic index <10 mitoses per 10HPF and no necrosis. Based on a variety of research methods, including RNA and DNA based NGS, first generation sequencing, FISH and nucleic acid gel electrophoresis, the *SLMAP-BRAF* fusion was fully validated. This fusion preserves C-terminal kinase domain (exons 11–18) and loses the N-terminal auto-inhibitory function domain (encoded by exons 1–8). Thus, this *BRAF* fusion form, similar to class II *BRAF* alterations, can produce an abnormal fusion product coupling the C-terminal BRAF kinase domain, leading to constitutive dimerization and activation of BRAF kinase activity (19). Ultimately, we made a descriptive diagnosis: *BRAF* rearrangement sarcoma with moderate-grade malignancy, non-specific type.

After hemimandibulectomy and the final diagnosis, chemotherapy (doxorubicin, ifosfamide and cisplatin regimen) was urgently initiated. The patient has finished six cycles of chemotherapy, and within 7 months of follow-up, no tumor recurrence or metastasis was observed. The tumor-free survival will be continually evaluated.

## Discussion

Sarcomas are a complex family of more than 70 different diseases deriving from bone and soft tissue (20). These rare tumors comprise 1-2% of adult malignant tumors worldwide (21). The diversity and rarity of sarcomas, coupled with lack of specific antibodies, bring the diagnosis under challenge, even leading to diagnostic errors up to 10-25% among pathological experts in sarcoma field (22, 23). NGS technologies have promoted tumor precision diagnosis and therapy over the last decade. Many molecular-targeted drugs are specific to gene translocations, especially to anaplastic lymphoma kinase (ALK) and tropomyosin receptor kinase (TRK) related gene fusion mutation (24).

Recently, *BRAF* fusions and targeted treatment have been reported successively. *BRAF* fusions are infrequent driver mutation, and limited to certain diseases including PA, spitzoid melanoma, pancreatic acinar carcinoma and papillary thyroid cancer (25). *BRAF* fusions occur extremely rare in sarcomas (0.2%) (26). Only IFS and MIFS have been reported to be enriched in *BRAF* driver fusions (8, 27). MEK inhibitors have been preliminarily proved to be efficacious for *BRAF* fusion tumor (11, 28). Therefore, the detection of *BRAF* fusion plays a vital role in understanding tumorigenesis and optimizing treatment.

Here, we report a case of mandible sarcoma with *BRAF* gene fusion and present detailed morphological and IHC features. The tumor was mainly composed of mononuclear cells, focally interspersed with a few spindle cells, forming special biphasic structure, accompanied with a few multinucleated giant cells. Histologically, tenosynovial giant cell tumor and giant cell tumor of bone must be considered in the differential diagnosis firstly. Tenosynovial giant cell tumor contains mononuelear cells with positive expression of clusterin, CD68 and Desmin, and also harbors CSF1 rearrangement at the genetic level. While in our case, CD68 showed positive expression in multinucleated giant cells rather than mononuelear cells (**Figure 3F**), and there were no positive findings of Clusterin and Desmin epression and CSF1 rearrangement through IHC and NGS detection separately. In addition, giant cell tumor of bone generally originates from the epiphysis of long bones, and H3F3A is found in 85% of giant cell tumors of the bone. Our case showed a mandible tumor with negative expression of H3G34W plus wildtype H3F3A gene result, so the diagnosis of giant cell tumor of bone is basically excluded. Osteosarcoma, as a relative common malignant tumor of bone, exhibits osteoblastic differentiation and produces malignant osteoid. Generally, osteosarcoma is characterized by highly complex chromosomal aneuploidy and intratumoral heterogeneity due to chromosomal instability. Somatic alterations involve various numerical and structural change. In this case, however, no identified neoplastic bone formation under careful observation, no other gene or chromosome alterations were found by 1166-panel RNA and 1238-panel DNA NGS detection as well, we explicitly eliminated the diagnosis of osteosarcoma, even though diffusely expression of SATB2. Although the histogenesis is still unknown, practically, nosology may be less important than identification of oncogenic genetic alterations potential to aid in diagnosis and treatment.

Other studies have demonstrated *BRAF* rearrangements involve in the *BRAF* kinase domain and various partners (9, 25). *SLMAP* is the first time to be reported as the partner gene of *BRAF* fusion. We are wondering whether the retained 11 exons in the N-terminal of *SLMAP* perform a function. Based on the existing research, it is speculated that the 5′ partner genes are uncorrelated with histological feature in IFS or MIFS (8, 9, 27). In our report, immunohistochemical stain of BRAF is weak, which is consistent with other’s findings: the mRNA expression levels of *BRAF* are not significantly increased in MIFS and IFS with *BRAF* rearrangement (8, 27). Therefore, immunohistochemical stain for BRAF protein is less likely to serve as a screening tool. In this jaw sarcoma, we see chronic inflammatory infiltration, the same phenomenon is also observed in MIFS (27) and part of IFS cases (8, 9). In a few cases, *BRAF* fusion related sarcomas harbor expression of S100 and/or CD34 (9, 29), but our case expressed neither S100 nor CD34. P16 has been reported as a senescence related protein, strongly expressing in degenerated or senescent cells (30). In our case, expression of p16 only appears in spindle cells, indicating spindle cells might be in senescence process. Moreover, negative or mild expression of Ki67 in these spindle cells is also in keeping with the degenerated phenomenon (not shown). It is regrettable that we haven’t found the identical morphological and immunohistochemical characteristics to help with diagnosis of this series of *BRAF* fusion sarcomas. *BRAF* fusion sarcomas represent heterogeneous diseases. No matter how the morphology varies, the detection of *BRAF* fusion has been increasingly worthwhile in guiding clinical treatment. A few clinical trials could be traced for BRAF inhibitor therapy. RAF inhibitors may be not effective against BRAF or CRAF fusions, however, the combination of RAF inhibitor and MEK inhibitor achieved unexpected clinical responses in some BRAF fusion cases (19). Although it is not clear how the combined drug therapy works, RAF and MEK double inhibitors may achieve the therapeutic effect in BRAF rearrangement sarcomas, especially when first-line treatment fails.

In conclusion, this is a rare case of sarcoma in the mandible with *SLMAP-BRAF* fusion demonstrating moderate-grade malignancy. We recognize *SLMAP* as a novel translocation partner in *BRAF* rearrangement sarcomas and provide researchers potential targeted treatment options for sarcomas involving this translocation.

## Data availability statement

The original contributions presented in the study are included in the article. Further inquiries can be directed to the corresponding author.

## Ethics statement

The studies involving human participants were reviewed and approved by the Ethics Committee of the Second Xiangya Hospital of Central South University. Written informed consent to participate in this study was provided by the participants’ legal guardian/next of kin. Written informed consent was obtained from the individual(s) for the publication of any potentially identifiable images or data included in this article.

## Author contributions

PZ: Writing – original draft. WL: Writing – review & editing, Methodology. JZ: Writing – review & editing, Data curation. HZ: Writing – review & editing. JL: Writing – review & editing, Funding acquisition.
